# Spatiotemporal Regulators for Insulin-Stimulated GLUT4 Vesicle Exocytosis

**DOI:** 10.1155/2017/1683678

**Published:** 2017-04-25

**Authors:** Xiaoxu Zhou, Ping Shentu, Yingke Xu

**Affiliations:** Department of Biomedical Engineering, Key Laboratory for Biomedical Engineering of Ministry of Education, Zhejiang Provincial Key Laboratory of Cardio-Cerebral Vascular Detection Technology and Medicinal Effectiveness Appraisal, Zhejiang University, Hangzhou 310027, China

## Abstract

Insulin increases glucose uptake and storage in muscle and adipose cells, which is accomplished through the mobilization of intracellular GLUT4 storage vesicles (GSVs) to the cell surface upon stimulation. Importantly, the dysfunction of insulin-regulated GLUT4 trafficking is strongly linked with peripheral insulin resistance and type 2 diabetes in human. The insulin signaling pathway, key signaling molecules involved, and precise trafficking itinerary of GSVs are largely identified. Understanding the interaction between insulin signaling molecules and key regulatory proteins that are involved in spatiotemporal regulation of GLUT4 vesicle exocytosis is of great importance to explain the pathogenesis of diabetes and may provide new potential therapeutic targets.

## 1. Introduction

GLUT4 is a 12-transmembrane facilitative glucose transporter that is primarily expressed in muscle and adipose tissues, where it is responsible for insulin-stimulated glucose disposal and for the entry of glucose to muscles during contraction and exercise (see latest reviews in [1–3]). The indispensible role of GLUT4 in regulation of glucose hemostasis has been well documented in the previous animal studies, in which genetic ablation of the *GLUT4* gene specifically in mice muscle or adipose tissues results in impaired glucose uptake, hyperinsulinemia, and peripheral insulin resistance [4, 5]. The major physiological action of insulin is to increase the glucose uptake and storage in muscle and adipose tissues, which is accomplished through the mobilization of intracellular GLUT4 storage vesicles (GSVs) to the cell surface upon stimulation. In the basal state, approximately 5–10% of the GLUT4 is located at the cell surface and >90% in intracellular membrane compartments. Insulin stimulation shifts the steady-state distribution of GLUT4 towards the plasma membrane. The dysfunction of insulin-stimulated GLUT4 translocation is highly related to peripheral insulin resistance and non-insulin-dependent diabetes mellitus in human beings [2].

Multiple insulin signaling pathways have been implicated in GLUT4 regulation, which may impinge on one or numerous steps along the intracellular itinerary of GLUT4 trafficking [6]. Although the insulin signal transduction network that controls GLUT4 translocation has been largely discovered (reviewed in [2, 7]), the mechanism of spatiotemporal coupling between the signaling and intracellular vesicle trafficking is still not fully understood. Insulin regulates GLUT4 vesicle exocytosis in a temporal and spatial manner. Insulin initiates rapid signaling transduction cascades that propagate into the cell to mobilize GLUT4 vesicle release. In addition, insulin promotes the spatial compartmentalization of signaling and protein machinery that play an important role in insuring the fidelity and specificity of its action on GLUT4 vesicle exocytosis. Here, we focus on the current understanding and recent work that have led to improved knowledge of how insulin signaling and key regulatory proteins are involved in spatiotemporal regulation of GLUT4 vesicle exocytosis.

## 2. Temporal Regulators of Insulin-Stimulated GLUT4 Translocation

Insulin stimulates the surface accumulation of GLUT4 with a half time of 2–5 minutes, which reaches a plateau after 12 minutes [8]. In this process, multiple trafficking steps of GLUT4 are potentially regulated by insulin signaling, including GSV release and trafficking [9], vesicle tethering/docking [10–12], and ultimately fusion [13–15].

**Figure 1:**
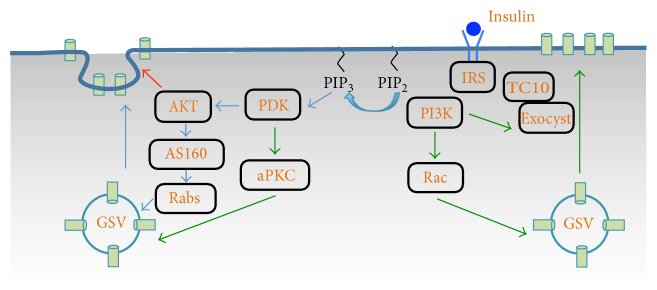
Schematic representation of insulin signaling pathways leading to GLUT4 vesicle exocytosis in muscle and adipose cells. See text for details.

Insulin signaling is initiated through binding and activation of its surface receptor. Activation of the insulin receptor triggers a cascade of phosphorylation events that ultimately promote GLUT4 vesicle exocytosis. The canonical insulin signaling pathway involves docking of the insulin receptor substrate (IRS) to the activated insulin receptor, which then subsequently activates phosphoinositide 3-kinase (PI3K). Activated PI3K increases the conversion of phosphatidylinositol 4,5-bisphosphate (PIP_2_) to phosphatidylinositol 3,4,5-trisphosphate (PIP_3_) at the plasma membrane, which activates Akt and atypical protein kinase C (aPKC) and subsequently phosphorylation of AS160 (Akt substrate of 160 kDa) by Akt (see reviews in [1, 7, 16]). In addition, a PI3K-independent pathway that is involved with c-CBL, c-CBL-associated protein (CAP), and the small GTPase TC10 may also regulate insulin-stimulated GLUT4 translocation in adipocytes (see reviews in [1, 17]). Together, these signaling pathways ensure the efficient delivery of GLUT4 to the cell surface by properly orchestrating lipids, protein kinases, small GTPase, and adaptor proteins at the plasma membrane (Figure 1).

### 2.1. PI3K

The PI3K-dependent insulin signaling plays a pivotal role in regulation of GLUT4 translocation in both muscle and adipose cells. There is a lot of evidence showing that PI3K activity is essential for insulin-stimulated GLUT4 translocation. Inhibition of PI3K activity with specific inhibitors, such as wortmannin and LY294002, or expression of dominant-negative mutants of PI3K and microinjection of blocking antibodies to PI3K can completely abolish insulin-stimulated GLUT4 translocation [18–20]. In contrast, overexpression of constitutively active form of PI3K [21–23] or exogenous addition of cell-permeable derivatives of PIP_3_ induces insulin-independent GLUT4 translocation [24]. Furthermore, a recent work from our group by using optogenetic approach (light-induced protein heterodimerization between CIBN-CAAX and CRY2-iSH2) to rapidly activate PI3K in adipocytes shows that light-activated PI3K fully mimics the effect of insulin on promoting GSV exocytosis [25]. Together, these studies demonstrate the central role of PI3K as a major effector in connecting insulin signaling with vesicle trafficking.

### 2.2. Akt/PKB

Akt/PKB, a serine-/threonine-specific protein kinase downstream of PI3K, is another crucial node in insulin signaling. There are three existing Akt isoforms (Akt1–3) in mammalian cells, and knockout and knockdown studies have identified Akt2 as the relevant isoform required for insulin-stimulated GLUT4 trafficking and glucose uptake [26–29]. Microinjection of Akt substrate peptide or antibody specific to Akt inhibits insulin-stimulated GLUT4 translocation to the plasma membrane by 66% and 56%, respectively, in 3T3-L1 adipocytes [30]. In L6 muscle cells, overexpression of Akt dominant-negative mutations decreases insulin-stimulated GLUT4 translocation by approximately 60% [31]. In addition, the drug inhibitor for Akt activation, Akti, does not fully abolish insulin-stimulated GLUT4 translocation in adipocytes [25, 32]. Using an optogenetic approach to control Akt activation, a study from our group has demonstrated that Akt only accounts for about two-third of a maximal insulin effect on GLUT4 translocation [25], which disagrees with the previous studies that claim Akt is sufficient for insulin-stimulated GLUT4 translocation [33–35]. Whether Akt and PI3K play equivalent roles in GLUT4 translocation needs to be further tested in other cell types or compared in a more physiological condition. However, it has been suggested that other PI3K signaling pathways, for instance, PI3K-activated Rac1 and aPKC pathways, are required together with Akt to promote GLUT4 translocation in muscle and adipose cells [36, 37].

### 2.3. AS160 and TBC1D1

AS160 (also known as TBC1D4) is a downstream effector of Akt/PKB, which has been shown to be a key modulator of intracellular GLUT4 translocation [38, 39]. AS160 is a Rab GTPase-activating protein (Rab-GAP), which is present on GLUT4 vesicles. Insulin causes the phosphorylation of AS160 at multiple serine/threonine residues, which inactivate its GAP activity. The current understanding is that in the basal state, the Rab-GAP function of AS160 promotes the inactive GDP-bound state of Rabs. However, in the presence of insulin stimulation, phosphorylation of AS160 shuts off its GAP activity, shifting the equilibrium of its target Rabs to an active GTP-bound state, which releases GLUT4 from intracellular retention mechanisms [38–40]. Indeed, studies have shown that knockdown of AS160 increases the localization of GLUT4 at the PM of unstimulated cells, which impairs insulin-stimulated GLUT4 translocation [38, 40]. Similarly, overexpression of Akt/PKB phosphorylation-deficient mutant of AS160, that is, constitutively active GAP, induces a reduction in Rab activity and insulin-stimulated GLUT4 translocation in both 3T3-L1 adipocytes and muscle cells [41, 42]. TBC1D1, another Rab-GAP protein that is highly homologous with AS160/TBC1D4, has shown to display similar regulation of GLUT4 in 3T3-L1 adipocytes and muscle cells [43, 44]. TBC1D1 is most abundant in the skeletal muscle and only has a very low abundance in adipocytes. Knockdown of endogenous TBC1D1 in 3T3-L1 adipocytes has no effect on insulin-stimulated GLUT4 translocation, whereas its overexpression diminishes the effect of insulin on GLUT4 translocation [43, 45]. Knockout of TBC1D1 protein expression in mice impairs exercise-mediated glucose uptake in muscle fibers and lowers GLUT4 expression but does not affect the fold change of insulin-stimulated glucose uptake in muscle [46, 47]. The latest animal studies by generating TBC1D1 Ser231Ala-knockin mice that are abortive of AMP-activated protein kinase- (AMPK-) induced phosphorylation show that TBC1D1 is more involved in AICAR-induced muscle glucose uptake and only partially mediates AMPK-regulated glucose homeostasis in muscle [48]. Nevertheless, this study has not measured phosphorylation of AS160 or TBC1D1 in response to exercise or AICAR on other sites besides Ser231, nor did it assess other means of Rab-GAP regulation by phosphorylation. Thus, the role of these two Rab-GAPs in different cells and in different stimulus conditions still deserves further studies in the field.

The identification and characterization of the downstream Rabs of AS160 and TBC1D1 have been an intense area of research. The previous in vitro experiments show that AS160 has Rab-GAP activity against Rabs 2, 8, 10, 13, and 14 [49, 50]. In adipocytes, the Rab proteins that are currently considered to be the main targets of AS160 are Rab10 and Rab14 [49, 51]. Studies show that knockdown of Rab10 diminishes insulin-stimulated GLUT4 translocation in 3T3-L1 adipocytes, whereas the surface recycling of a transferrin receptor is unaffected, suggesting that Rab10 can act specifically at the GSVs [51, 52]. To support this, knockdown of Dennd4C, the guanine nucleotide exchange factor (GEF) for Rab10, inhibits insulin-stimulated GLUT4 translocation in adipocytes [53]. On the contrary, studies suggest that Rab14 is involved in the endosomal recycling of GLUT4, probably engaging in intracellular sorting of GLUT4 into GSVs [51, 54, 55]. In muscle cells, it has been shown that Rab8 and Rab13 are the major targets of AS160, whose downregulation profoundly inhibits insulin-stimulated GLUT4 translocation [56–59]. In addition, other Rabs that are implicated in intracellular GLUT4 traffic include Rab4 [60], Rab5 [61], Rab11 [62], Rab28 [63], and Rab31 [64]. On the other hand, the Rabs associated with exercise-stimulated GLUT4 traffic are still unknown. The Rabs may work collectively to ensure the proper traffic of GLUT4 through the intracellular compartments to the PM.

### 2.4. aPKC

Atypical PKCs (including protein kinase *ζ* and *ι*/*λ* isoforms), which belong to the PKC family, require neither calcium nor diacylglycerol for activation. Blocking the activation of PKC*λ* partly inhibits GLUT4 trafficking [65, 66]. Overexpression of dominant-negative mutants of PKC*λ* inhibit insulin-stimulated glucose uptake by ~60%, and these mutants do not inhibit insulin-induced activation of Akt [65]. In addition, muscle-specific knockout of PKC*λ* has shown to induce systemic insulin resistance and diabetes in mice, which further demonstrates the importance of aPKC in insulin-stimulated glucose transport [67]. On the contrary, overexpression of constitutively active PKC*λ* or PKC*ζ* isoforms have been demonstrated to promote GLUT4 translocation in 3T3-L1 adipocytes [65], rat skeletal muscle cells [68], and primary rat adipocytes [69]. Interestingly, studies in muscle cells show that activation of PKC induces serine phosphorylation of VAMP2 in the GLUT4 compartment, which subsequently increases glucose transport [70]. Another research in adipocytes indicates that aPKC*ζ*/*λ* is a convergent downstream target of the insulin-stimulated PI3K and TC10 signaling pathways [36]. Furthermore, the exocyst subunit Sec5 has shown to be regulated by PKC signaling, which modulates the stability of exocyst complex through phosphorylation modification [71]. However, the downstream effectors of PKC and the underlying mechanism of regulation in GLUT4 translocation are still needed in the field. The connection of aPKC signal to GLUT4 vesicle trafficking appears to involve the actin cytoskeleton, Rabs, and molecular motors, as PKC*λ*/*ζ* can impinge on Rac-mediated actin dynamics and can also regulate the interaction between Rab4 and kinesin motors [72, 73].

### 2.5. Rac and TC10

Insulin-stimulated GLUT4 translocation requires dynamic changes in the actin cytoskeleton or called actin remodeling. Remodeled actin may serve as a scaffold that directs selective signaling molecules for proper signal transduction or alternatively may serve as tracks for motor proteins to move GLUT4 vesicles to the PM. These functions of regulation appear to be engaged with small G protein activity: Rac1 in muscle cells and TC10 in adipocytes.

Rac is one of the Rho GTPase family members. Rac1 is the only isoform that is shown to be involved in insulin-stimulated GLUT4 translocation in muscle cells [74]. In muscle-specific Rac1 knockout mice, both insulin- and exercise-stimulated GLUT4 translocations and glucose uptake are markedly impaired [74, 75]. Similarly, in L6 cells with Rac1 knockdown or overexpression of dominant-negative mutant of Rac1, the increase in insulin-stimulated GLUT4 translocation is fully abolished [76, 77]. Under these circumstances, insulin-stimulated actin remodeling is affected, presumably through the downregulation of Rac1 activity. In muscle cells, it has been suggested that Akt and Rac1 are two parallel signaling pathways under the regulation of PI3K [74, 77–79]. Rac1 knockdown or constitutive activation has no effect on insulin-stimulated Akt phosphorylation [77, 79]. On the contrary, some other studies suggest that Akt signaling is an upstream of Rac1 in skeletal muscle cells [80, 81]. These studies show that insulin-induced Rac1 activation is completely inhibited by Akt inhibitors or with Akt2 knockdown in muscle cells [80, 81]. Thus, the mechanism of Rac1 activation and its potential role in regulation of GLUT4 trafficking in adipose cells are yet to be established.

TC10 is a member of the Rho small GTPase known to regulate cortical actin dynamics and contribute to GLUT4 exocytosis [82, 83]. In adipocytes, TC10 becomes activated in response to insulin stimulation, and this activation appears to be PI3K-independent, under the regulation of the CAP/Cbl/C3G cascade of the signaling pathway (see reviews in [2, 17, 84]). TC10 has homology sequence with Cdc42 and Rac and binds to effectors having a Cdc42-/Rac-interactive binding domain, such as p21-activated protein kinase, the neural Wiscott-Aldrich syndrome protein (N-WASP) [85], and also proteins without these domains, for instance, Exo70 [86], PIST [87], and CIP4 [88]. In 3T3-L1 adipocytes, overexpression of dominant-interfering TC10alpha mutant inhibits insulin-stimulated glucose uptake and GLUT4 translocation [82, 89], suggesting the importance of GTPase activity in its function. On the contrary, another study shows that overexpression of TC10alpha or other chimeras with lipid raft-targeting motifs in adipocytes inhibits insulin-stimulated GLUT4 translocation, and these effects are independent of its GTPase activity but dependent on its membrane localization [90]. Furthermore, although siRNA-mediated TC10 knockdown was reported to effect insulin-simulated GLUT4 translocation, no other lab has reported the same findings and no mouse knockout models have corroborated these findings found in 3T3-L1 adipocytes. In addition, in muscle cells, TC10 mutations are shown to fail to prevent insulin-stimulated GLUT4 translocation [76]. Thus, additional studies are needed to elucidate the role of TC10 in regulation of the trafficking of GLUT4, especially in muscle systems, and how these intracellular effectors of TC10 regulate discrete steps of GLUT4 trafficking in cells.

## 3. Spatial Determinants of Insulin-Regulated GLUT4 Translocation

The spatial aspects of insulin signal transduction and distribution of regulatory proteins play a crucial role in determining the specificity of insulin action. So far, lots of insulin signaling molecules, such as insulin receptor [91], CAP, and its interacting proteins [89, 92], have been found to associate with or reside on the subdomain of the plasma membrane lipid raft structure. The role of lipid rafts in insulin signaling has been reviewed elsewhere [17, 93]. Here, we mainly focus on the description of membrane traffic regulatory proteins in spatial regulation of GLUT4 vesicle exocytosis.

### 3.1. Exocyst Complex

An exocyst is an evolutionarily conserved octameric protein complex consisting of Sec3, Sec5, Sec6, Sec8, Sec10, Sec15, Exo70, and Exo84 (see reviews in [94, 95]). The exocyst components were originally identified in a genetic screen for temperature-sensitive secretory mutants of yeast *Saccharomyces cerevisiae* [96]. The exocyst complex plays a crucial role in the targeting of vesicles to the plasma membrane during exocytosis, and it has been shown to be involved in diverse cellular processes, such as yeast budding, cell polarity, ciliogenesis, and neurite outgrowth (see reviews in [94, 95]). The exocyst has been demonstrated to play a pivotal role in insulin-stimulated GLUT4 trafficking, presumed by facilitating the tethering/docking of GLUT4 vesicles to the plasma membrane [86, 97]. It has been shown that the GTPase TC10 interacts with Exo70 and recruits Exo70 and Sec6/Sec8 subcomplex components to the cell surface after insulin stimulation [86]. Overexpression of dominant-negative mutant of Exo70 blocks insulin-stimulated GLUT4 vesicle fusion with the plasma membrane but not the redistribution of vesicles to the periphery of cells [86]. In 3T3-L1 adipocytes, studies have shown that insulin stimulation promotes the redistribution of Sec6 and Sec8 to the cell surface, and overexpression of Sec6/Sec8 exocyst subunits augments insulin-stimulated GLUT4 translocation [97]. In addition, Sec8 interacts with synapse-associated protein 97 (SAP97) in lipid rafts, which anchors the exocyst complex in the subdomain of the plasma membrane [98]. Besides, both Sec3 and Exo70 can interact with PIP_2_, the phospholipid present in lipid rafts through PH domain-like structure [99, 100]. Thus, it is conceivable that exocyst complex assembles in subdomains at the plasma membrane, which regulates the spatial localization and fusion of GLUT4 with the plasma membrane. Indeed, the previous work has shown that the exocytic sites of insulin-stimulated GLUT4 vesicle exocytosis are spatially clustered on the plasma membrane, which are disrupted and become randomized after Sec8 knockdown [101]. Together, these studies suggested that the exocyst might serve as a spatial landmark for GLUT4 vesicle exocytosis at the plasma membrane. In the future, direct visualization of exocyst dynamics and GLUT4 vesicle exocytosis is needed to better illustrate this point.

### 3.2. SNARE Proteins

The primary role of SNAREs (soluble N-ethylmaleimide-sensitive factor attachment protein receptors) is to bridge two membranes and drive membrane fusion events, which control the membrane traffic in all eukaryotic cells [102]. The interaction between SNARE proteins from the vesicle (v-SNAREs) and those from the target membrane (t-SNAREs) is essential for membrane fusion. The SNARE proteins involved in the fusion of GLUT4 vesicles with the plasma membrane are syntaxin 4, SNAP23, and VAMP2 [103, 104]. The t-SNAREs syntaxin 4 and SANP23 are located on the plasma membrane, which mark the exocytic sites where GLUT4 vesicles fuse. It has been reported that syntaxin 4 and SNAP23 are not uniformly distributed on the cell surface. Syntaxin 4 and SNAP23 reside on cholesterol-enriched lipid raft structure which occupies discrete areas on the plasma membrane [105, 106]. It has been generally accepted that VAMP2 is the v-SNARE present on GSVs, although other v-SNAREs, such as VAMP3, VAMP7, and VAMP8, have also been suggested to be present on GLUT4 vesicles and implicated in vesicle exocytosis [14, 107, 108]. Similar to syntaxin 4 and SNAP23, VAMP2 has also been demonstrated to localize in lipid rafts [106]. Together, these studies strongly suggest the lipid rafts play an important role in the process of insulin-stimulated GLUT4 vesicle exocytosis at the plasma membrane.

In addition, proteins that can regulate SNARE function may engage in spatial regulation of vesicle exocytosis. One particular interesting protein is Munc18c, which is a member of Sec1/Munc18 (SM) family proteins. SM proteins are essential regulators of SNARE-mediated vesicle fusion events, initially identified as high-affinity binding partners for syntaxin proteins on the plasma membrane and, more recently, in a binding mode with the SNARE core complex [109]. In adipocytes, Munc18c interacts with syntaxin 4. Overexpression of Munc18c has shown to inhibit insulin-stimulated GLUT4 translocation, but in adipocytes derived from MEFs with Munc18c knockout, it shows enhanced GLUT4 translocation [110, 111]. These studies suggest that disruption of the interaction between syntaxin4 and Munc18c might serve another function of insulin regulation. Indeed, researches have demonstrated that insulin signaling through the insulin receptor kinase regulates the assembly of SNARE complexes by controlling the phosphorylation of Munc18c [112, 113]. In the basal state, Munc18c interacts with syntaxin 4, which blocks the availability of syntaxin 4 to interact with VAMP2 and t-SNAREs. Whereas in the presence of insulin, insulin receptor tyrosine kinase phosphorylates Munc18c on Tyr219 and Tyr521 sites, which release Munc18c from syntaxin4, thus promotes the SNARE complex formation, and increases insulin-stimulated GLUT4 vesicle exocytosis [112, 113].

## 4. Perspectives

To dissect the spatiotemporal relationship between insulin signaling, protein dynamics, and GLUT4 vesicle trafficking, new techniques are greatly needed in this field. For instance, new approaches to perturb insulin signaling in a rapid and specific manner, our group has recently applied an optogenetic system to control the activation of PI3K and Akt both spatially and temporally and dissect the role of individual of them in insulin-stimulated GLUT4 vesicle exocytosis [25]. Integration of optogenetics with high-resolution light microscope imaging and the dynamic function of signaling nodes in vesicle trafficking that is not easily targetable with drugs can be visualized and analyzed. In addition, a large-scale and high-throughput proteomics study is needed. Although the insulin signaling pathway and key molecular players have been identified and characterized, the intersection of different signaling pathways and new components that are potentially involved in regulation of vesicle trafficking remains to be discovered.
